# The Generation of Successive Unmarked Mutations and Chromosomal Insertion of Heterologous Genes in *Actinobacillus pleuropneumoniae* Using Natural Transformation

**DOI:** 10.1371/journal.pone.0111252

**Published:** 2014-11-19

**Authors:** Janine T. Bossé, Denise M. Soares-Bazzolli, Yanwen Li, Brendan W. Wren, Alexander W. Tucker, Duncan J. Maskell, Andrew N. Rycroft, Paul R. Langford, on behalf of the BRaDP1T Consortium

**Affiliations:** 1 Section of Paediatrics, Imperial College London, St Mary's Campus, London, United Kingdom; 2 Laboratório de Genética Molecular de Micro-organismos, Departamento de Microbiologia - DMB – BIOAGRO, Universidade Federal de Viçosa – Viçosa, Brazil; 3 Department of Pathogen Molecular Biology, London School of Hygiene and Tropical Medicine, London, United Kingdom; 4 Department of Veterinary Medicine, University of Cambridge, Cambridge, United Kingdom; 5 Department of Pathology and Pathogen Biology, The Royal Veterinary College, North Mymms, Hatfield, United Kingdom; The University of Melbourne, Australia

## Abstract

We have developed a simple method of generating scarless, unmarked mutations in *Actinobacillus pleuropneumoniae* by exploiting the ability of this bacterium to undergo natural transformation, and with no need to introduce plasmids encoding recombinases or resolvases. This method involves two successive rounds of natural transformation using linear DNA: the first introduces a cassette carrying *cat* (which allows selection by chloramphenicol) and *sacB* (which allows counter-selection using sucrose) flanked by sequences to either side of the target gene; the second transformation utilises the flanking sequences ligated directly to each other in order to remove the *cat*-*sacB* cassette. In order to ensure efficient uptake of the target DNA during transformation, *A. pleuropneumoniae* uptake sequences are added into the constructs used in both rounds of transformation. This method can be used to generate multiple successive deletions and can also be used to introduce targeted point mutations or insertions of heterologous genes into the *A. pleuropneumoniae* chromosome for development of live attenuated vaccine strains. So far, we have applied this method to highly transformable isolates of serovars 8 (MIDG2331), which is the most prevalent in the UK, and 15 (HS143). By screening clinical isolates of other serovars, it should be possible to identify other amenable strains.

## Introduction

Porcine pleuropneumonia, caused by *Actinobacillus pleuropneumoniae*, is an endemic disease that continues to cause considerable economic losses in the swine industry worldwide [1], [2]. After good husbandry practices are taken into account, there are two basic methods used to limit endemic infection: vaccines and antibiotics. Increasing resistance to antibiotics limits their efficacy, and there is growing pressure against the use of antibiotics in livestock production. Therefore development of an effective vaccine is required for control of this important disease.

Although bacterin (killed whole cell) and subunit vaccines have been developed for *A. pleuropneumoniae*, none has conferred complete protection against infection with all serovars (for a review, see [3]). There is growing interest in development of live attenuated vaccines (LAVs), as they have the potential to protect against homologous and heterologous serovars [4]–[6]. For licensing purposes, a LAV should not contain antibiotic resistance markers, and ideally should be easily differentiated from clinical isolates [5]–[7]. Furthermore, an ideal LAV for *A. pleuropneumoniae* might also be used as a vector for heterologous protection against other pig pathogens.

At present, the only system for introducing unmarked mutations into *A. pleuropneumoniae* is based on the use of suicide vectors (pBMK1 and pEMOC2) carrying the counter-selectable *sacB* gene [8], [9]. First developed and most widely used in serovar 7 strain AP76 [8]–[17], it has been successfully applied to selected strains of serovars 1, 2, and 5 [6], [7], [18]. However, this system does not work in all strains [7]. Because of the nature of the system, which involves co-integration of the vector and formation of a merodiploid, upon counter-selection, resolution of the integrated plasmid can result either in the strain retaining the mutated copy of the target gene or in a return to the wild-type genotype. Although there should be an equal likelihood of either result, this is not always the case, and detection of the desired mutant strain may require screening of large numbers of colonies.

We have previously reported that some strains of *A. pleuropneumoniae* are capable of natural transformation [19], [20]. The reference strains of serovars 1, 3, 4, 5 and 8 all showed low frequencies of transformation (10^−8^–10^−9^), whereas the serovar 15 reference strain, HS143 [21], had a transformation frequency of 10^−4^ [20]. Despite the low transformation frequency of the serovar 1 reference strain, Shope 4074, we and others, have used natural transformation for generation of insertion-deletion mutations [19], [22]–[25]. Here we describe a simple two-step transformation system using linear DNA for generation of unmarked mutations in highly transformable isolates of *A. pleuropneumoniae*.

## Materials and Methods

### Bacterial strains and growth conditions

*Escherichia coli* XL1-Blue (Stratagene) or Stellar (Clontech), used for plasmid construction, were propagated on Luria-Bertani (LB; Difco) agar or in LB broth supplemented, when necessary, with 20 µg/ml chloramphenicol (Cm) or 100 µg/ml ampicillin (Amp). *A. pleuropneumoniae* serovar 8 (UK clinical isolates, including MIDG2331) and serovar 15 (reference strain, HS143) were grown at 37°C in 5% CO_2_ on brain heart infusion agar (BHI; Difco) supplemented with 0.01% β-nicotinamide adenine dinucleotide (BHI-NAD) or in BHI-NAD broth. When required, 1 µg/ml Cm was added for selection of transformants. For sucrose counter-selection, bacteria were plated onto salt-free LB agar consisting of 10 g tryptone, 5 g yeast extract, and 1.5 g agar per L supplemented with 10% filter-sterilised sucrose (LB-S) for *E. coli* clones, or onto salt-free LB agar supplemented with 10% sucrose, 10% horse serum and 0.01% NAD (LB-SSN) for *A. pleuropneumoniae* clones.

### DNA manipulations

Genomic DNA was prepared from bacterial strains using a QIAamp mini DNA kit, and plasmid extractions were performed using Qiaprep spin columns (Qiagen), according to the manufacturer's protocols. DNA concentrations were measured using a NanoDrop ND-1000 UV-Vis Spectrophotometer (NanoDrop Technologies). Unless otherwise stated, restriction enzymes were obtained from Roche and used according to the manufacturer's protocol. PCR was performed using either the QIAGEN Fast Cycling PCR Kit (Qiagen) or the CloneAmp HiFi PCR Premix (Clontech), according to the manufacturers' protocols.

### Identification of highly transformable serovar 8 isolate(s)

In order to identify more highly transformable isolates of serovar 8 of *A. pleuropneumoniae*, we tested 15 UK clinical isolates (collected between 1992 and 2003 from different parts of the UK) by the plate transformation assay previously described [19]. Briefly, individual isolates were grown in BHI-NAD broth to an OD_600_ of approximately 0.5, and 10 µl were spotted in duplicate onto BHI-NAD agar (8 spots per plate). Strain HS143 (serovar 15 reference strain), previously shown to be highly transformable [20], was used as a positive control. Following 100 min incubation at 37°C in 5% CO_2_, 750 ng of marked genomic DNA (serovar 15 *sodC*::Cm) were added to one spot of each strain (10 µl of 75 ng/µl), and cultures incubated for a further 4 h. Using a 1 µl loop, a small amount of culture was removed, bisecting each spot, and streaked for isolated colonies on BHI-NAD-Cm. The selection plates were incubated overnight at 37°C in 5% CO_2_. Strains resulting in good growth on BHI-NAD-Cm plates were tested further to determine transformation frequency, as previously described [19].

### Construction of the counter-selectable cassette

A 2.1 kb sequence containing the *omlA* promoter and *sacB* gene was amplified by PCR from pBMK1 [8], a generous gift from Professor Gerald-F. Gerlach, using primers sacB_For and sacB_Rev (see Table 1 for all primers used in this study), which added ApaI sites on both ends of the amplicon. The PCR product was digested with ApaI, cleaned using a Qiaquick spin column (Qiagen), and ligated using T4 DNA ligase (New England Biolabs) into ApaI-digested pUSScat vector (a pGEMT plasmid containing an 842 bp insert comprised of a *cat* gene flanked by 2 copies of the uptake signal sequences (USS) required for natural transformation in *A. pleuropneumoniae* [23], [26]), which was dephosphorylated using Shrimp Alkaline Phosphatase (Roche). The ligation mix was transformed into *E. coli* XL1-Blue cells (Stratagene). Transformants were selected on LB-Cm, and screened by colony PCR for the presence of the *sacB* gene. Sucrose sensitivity of selected clones was confirmed by patching onto LB-S. Restriction mapping of the pUSScatsac plasmid confirmed the insertion of the *sacB* gene downstream of, and in the same orientation as, the *cat* gene.

**Table 1:** Primers used in this study.

Name	Sequence
sacB_for	GCGTAATACGACTCACTATAGGGCCCATTG
sacB_rev	TTCCGCTTCCTTTAGGGGCCCTTG
catsacB_for	GATTCGCGGATCCGAGCTCTCTAAC
catsacB_rev	GCGTGAAGCTCGAGGTATGGGATTC
sodCleft_for	GGATTCGCCAAT*aCCGCTTGt*ACG
sodCright_rev	CCTTATTAAATGGCGGACCGACTTTCC
sodCcat_left	**TCGGATCCGCGAATC** GATGCGCCGAATAATGTAAAAGCAAGAG
sacBsodC_right	**CCTCGAGCTTCACGC** GGCTTGCGGCGTCATCAAATAGC
deltasodC_left	**ATGACGCCGCAAGCC** GATGCGCCGAATAATGTAAAAGCAAGAG
deltasodC_right	**ATTATTCGGCGCATC** GGCTTGCGGCGTCATCAAATAGC
ureCleft_for	CGGTCATAA*aCAAGCGGT*CTATTTTCAG
ureCright_rev	GATTGTGCCGATATTGAGTTCTGTACCAAAC
ureCcat_left	**TCGGATCCGCGAATC** CCATTTTCTGCCCCCTATAATTTGC
sacBureC_right	**CCTCGAGCTTCACGC** CGTGTGGACGGCGAGCATATTACTTG
deltaureC_left	**CTCGCCGTCCACACG** CCATTTTCTGCCCCCTATAATTTGC
deltaureC_right	**GGGGGCAGAAAATGG** CGTGTGGACGGCGAGCATATTACTTG
ureCnadVleft	**GGGCTCGGTTACTAG** CCATTTTCTGCCCCCTATAATTTGC
nadVureC_right	**ACTCGTGCGGCCGCC** CGTGTGGACGGCGAGCATATTACTTG
nadV_for	CTAGTAACCGAGCCCGCCTAATGAG
nadV_rev	GGCGGCCGCACTAGTGATTACAAG

ApaI sites in the sacB_for and sacB_rev primers are underlined. The USS present in sodCleft_for and ureCleft_for are indicated in italics with the lower case letters indicating a base change from the native sequence in order to generate a USS. The 15-bp extensions required for In-Fusion cloning are indicated in bold text.

### Deletion of *sodC* and/or *ureC*

**Figure 1:**
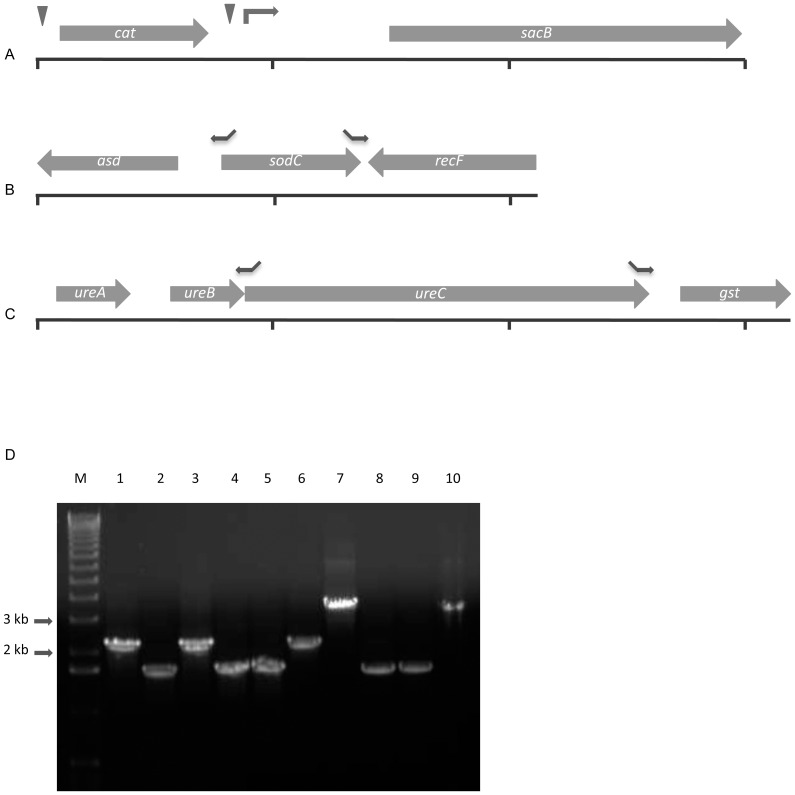
Construction and PCR verification of *sodC* and *ureC* deletions. A) Map showing the 3.0 kb *cat-sacB* cassette amplified from pUSScatsac using using catsacB_for and catsacB_rev. Triangles above the map indicate positions of the 2 USS required for efficient transformation, the bent arrow indicates the position of the *omlA* promoter. B) Map showing 2.1 kb sequence amplified using sodCleft_for and sodCright_rev (cloned into pTsodCF). Arrows above the map indicate positions of primers used in inverse PCR to delete a 504 bp region of *sodC*, and to add 15-bp overhangs required for fusion to the *cat-sacB* cassette (sodCcat_left and sacBsodC_right) or for direct fusion of the left and right flank sequences (deltasodC_left and deltasodC_right). C) Map showing the 3.2 kb sequence amplified using ureCleft_for and ureCright_rev (cloned into pTureCF). Arrows above the map indicate positions of primers used in inverse PCR to delete a 1641 bp region of *ureC*, and to add 15-bp overhangs required for fusion to the *cat-sacB* cassette (ureCcat_left and sacBureC_right), for direct fusion of the left and right flank sequences (deltaureC_left and deltaureC_right), or fusion to a 1914 bp fragment containing the *nadV* gene from *H. ducreyi* (ureCnadVleft and nadVureC_right). D) PCR amplification using primers sodCleft_for and sodCright_rev (lanes 1–6) or ureCleft_for and ureCright_rev (lanes 7–10) with template DNA from: 1) sero 15 WT; 2) sero 15 Δ*sodC*; 3) sero 8 WT; 4) sero 8 Δ*sodC*; 5) sero 8 Δ*sodC*Δ*ureC*; 6) sero 8 Δ*ureC*; 7) sero 8 WT; 8) sero 8 Δ*ureC*; 9) sero 8 Δ*sodC*Δ*ureC*; 10) sero 8 Δ*ureC*::*nadV*. M = 1 kb DNA ladder (Invitrogen).

The primers used in creation of the constructs are shown in Table 1. Where required, 15 bp extensions were added to the 5′ end of primers to allow directional cloning of the PCR fragments using the In-Fusion kit (Clontech) according to the manufacturer's protocol. The 3 kb *cat-sacB* cassette (Figure 1A) was amplified from pUSScatsac using primers catsacB_for and catsacB_rev. As mentioned above, this cassette contains 2 copies of the USS to facilitate natural transformation in *A. pleuropneumoniae.* Flanking sequences for the gene deletions were amplified from MIDG2331 chromosomal DNA using appropriate primer pairs. In cases where the amplified *A. pleuropneumoniae* sequences did not contain native USS, these were engineered into primers so that the deletion constructs would be efficiently taken up in the second transformation step. All fragments for the *cat-sacB* insertion and deletion constructs were amplified using proof-reading CloneAmp HiFi PCR Premix. Initially, PCR amplicons containing the genes to be deleted (Figures 1B and 1C), flanked by at least 600–1000 bp to either side, were cloned into pGEMT (Promega) to create pTsodCF and pTureCF. Inverse PCR was then used to open up the vectors, removing the target sequence and adding 15 bp overhangs to allow insertion of the *cat-sacB* cassette by In-Fusion cloning. The resulting In-Fusion products were transformed into *E. coli* Stellar cells (Clontech) and were selected on LB agar containing 20 µg/ml Cm, as required. PCRs were performed using the QIAGEN Fast Cycling PCR Kit (Qiagen) on selected colonies in order to confirm the presence of inserts. Selected *cat-sacB*-containing clones were confirmed as being sensitive to sucrose by patching onto LB-S plates. The deletion constructs were generated by amplifying the left and right flanking sequences with added 15 bp overhangs designed to allow direct fusion by overlap-extension (OE) PCR. For example, the *sodC* flanking regions were amplified using the primer pairs sodCleft_for/deltasodC_left and deltasodC_right/sodCright_rev. The resulting amplicons were combined, diluted 1/100, and used as template for OE-PCR using the primer pair sodCleft_for/sodCright_rev. The resulting deletion constructs were cloned into pGEMT.

Gene knockouts were achieved by two sequential transformation steps. In the first step, the plasmids containing the *cat-sacB* cassette flanked by *A. pleuropneumoniae*-specific sequence were linearised with NotI and transformed into the different *A. pleuropneumoniae* strains by natural transformation on agar plates, as previously described [19]. Cm-resistant transformants were screened for the appropriate insertion-deletion by PCR, and were tested for sensitivity to sucrose on LB-SSN. Subsequently, deletion constructs (either purified OE-PCR products, or linearised pGEMT clones containing the OE-PCR products) were used to transform appropriate insertion-deletion mutants in order to remove the *cat-sacB* cassette. Transformants were plated on LB-SSN, and sucrose-resistant transformants were screened for Cm sensitivity on BHI-NAD-Cm. Selected Cm-sensitive clones were tested by PCR to confirm the appropriate deletion. The double mutant (serovar 8 Δ*sodC*Δ*ureC*) was obtained by transformation of the serovar 8 Δ*sodC* mutant with linearised pTΔ*ureC*::*catsacB* construct, followed by removal of the *cat-sacB* cassette using the linearised pTΔ*ureC* construct in a second transformation. Loss of urease activity was confirmed by addition of urea base medium (Difco) to overnight broth cultures, as previously described [27]. Loss of SodC was confirmed by dot blot using the monoclonal antibody HD1, as previously described [28].

### Replacement of *ureC* with the *Haemophilus ducreyi nadV* gene

A 1.5 kb sequence containing the *nadV* gene was amplified from *H. ducreyi* genomic DNA (using primers listed in Table 1) and was directionally cloned into the appropriate inverse PCR product of pTΔ*ureC*F (amplified using ureCnadV_left and nadVureC_right as primers) using the In-Fusion kit. Following transformation into *E. coli* Stellar cells, clones were screened by PCR to identify the correct insertion. Plasmid prepared from a selected clone was linearised with NotI prior to use as template DNA to transform the sero8Δ*ureC*::*catsacB* mutant in order to remove the *cat-sacB cassette*. Sucrose resistant colonies were screened for Cm-sensitivity and the ability to grow on BHI without addition of NAD.

## Results

### Identification of a highly transformable serovar 8 isolate

Of the 15 UK clinical isolates tested, we identified one that had a transformation frequency of 1.9×10^−5^ (serovar 8 strain MIDG2331). This transformation frequency is at least 3 logs greater than previously shown for the serovar 8 reference strain [20].

### The unmarked mutation system

The *cat-sacB* cassette (Figure 1A) facilitated generation of multiple successive mutations in *A. pleuropneumoniae* using the two-step transformation protocol. Cm selection was very stringent, and all Cm-resistant clones tested were confirmed to contain the *cat-sacB* cassette by PCR (data not shown). Following counter-selection after the second round of transformation, spontaneous resistance to sucrose was evident, but the high transformation frequencies for HS143 and MIDG2331 (10^−4^ to 10^−5^ for each transformation) made it possible to isolate transformants and confirm the deletion by PCR.

### Deletion of *sodC* and/or *ureC*

**Figure 2:**
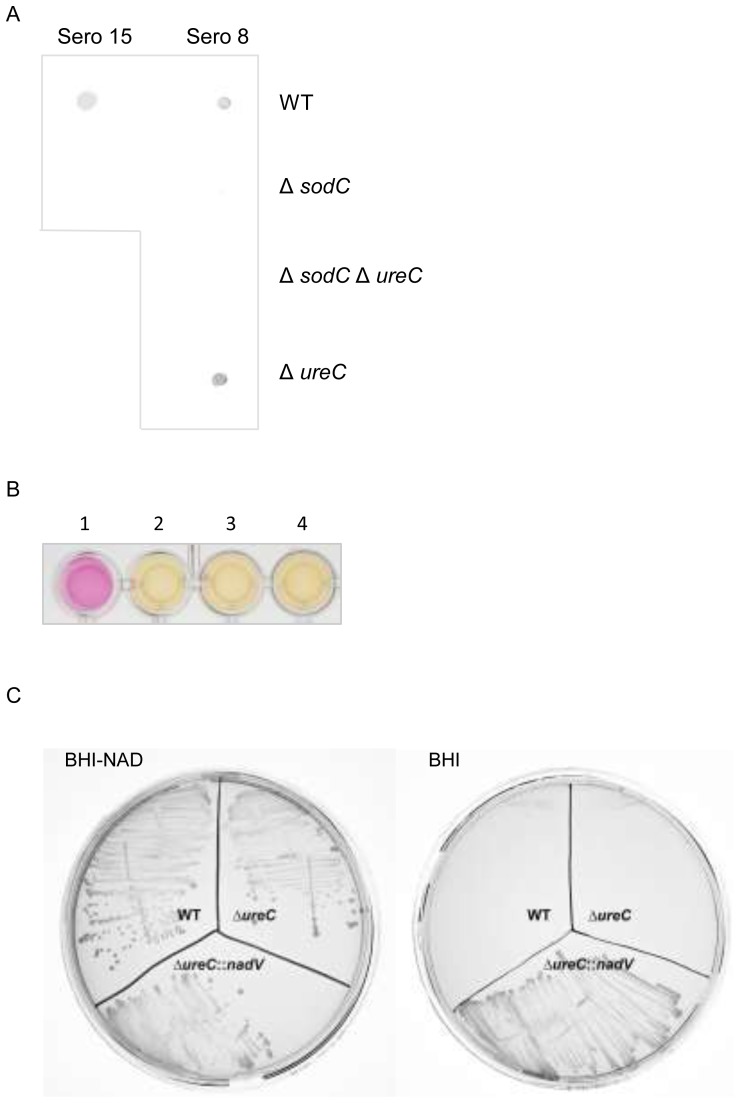
Phenotypic confirmation of mutations. A) Detection of SodC. Whole cell lysates (10 µg protein) were tested for reactivity with mouse monoclonal antibody HD1 by dot blot. B) Detection of urease activity in broth cultures of 1) sero 8 WT; 2) sero 8 Δ*ureC*; 3) sero 8 Δ*sodC*Δ*ureC*; and 4) sero 8 Δ*ureC*::*nadV*. A change in colour from yellow to pink indicates a positive reaction for urease activity. C) Growth of sero 8 strains on BHI-NAD and BHI (no NAD).

As proof of principle, the two-step transformation system was used to generate unmarked mutations of *sodC* and/or *ureC* in serovars 8 and 15 of *A. pleuropneumoniae*. These genes (encoding a [Cu,Zn]-superoxide dismutase and a subunit of the urease enzyme, respectively) were chosen because they are present in all *A. pleuropneumoniae* serovars and have easily detectable phenotypes. Deletion of *sodC* and/or *ureC* was confirmed in the different strains as shown by PCR (Figure 1D), SodC dot blot (Figure 2A), and urease activity assay (Figure 2B), as appropriate.

### Replacement of *ureC* with the *H. ducreyi nadV* gene

In order to illustrate the usefulness of this method for introducing foreign genes into targeted locations in the chromosome of *A. pleuropneumoniae*, a portion of the *ureC* gene was replaced with the *H. ducreyi nadV* gene. The *nadV* gene was chosen as heterologous expression from a plasmid was previously shown to result in NAD-independence in *A. pleuropneumoniae* [29], making it easy to phenotypically verify the insertion following sucrose counterselection. Expression of the chromosomally inserted *nadV* gene rendered *A. pleuropneumoniae* strains NAD-independent (Figure 2C), while elimination of urease activity in the mutant (Figure 2B), along with PCR verification (Figure 1D), confirmed the targeted location of the insertion.

## Discussion

The introduction of unmarked mutations into the bacterial chromosome is particularly desirable for generation of multiple mutations and LAVs. In *A. pleuropneumoniae,* suicide vectors (pBMK1 and pEMOC2) have been used by some groups to generate (multiple) unmarked mutations in selected strains [5], [6], [7], [18]. In our experience, however, although co-integrates are readily selected following conjugation of constructs based on these plasmids, counter-selection on sucrose tends to yield high numbers of wild-type revertants, making identification of true deletion mutants extremely laborious and often impossible.

Recently, a markerless mutation system was described for *Actinobacillus succinogenes* [30]. The method used a combination of natural transformation for introduction of insertion/deletion mutations with FRT sites flanking the selective marker, and electroporation with a plasmid expressing the Flp recombinase to drive excision of the marker. This method leaves a residual FRT site (scar) with each deletion, and requires curing of the plasmid expressing the recombinase. Although multiple successive mutations are possible, the build up of FRT scars in the chromosome could lead to recombination hotspots, which would not be desirable in a LAV strain.

Previously, we have shown the utility of natural transformation for generation of insertion-deletion mutations in *A. pleuropneumoniae* using linear dsDNA constructs [19], [23], [25]. By adding a counter-selectable gene into the insertion-deletion cassette and a second transformation step to remove the cassette, we have further exploited this simple technique to generate scarless unmarked deletions, and to insert a heterologous gene into a targeted site in the chromosome. This method can also be used for the generation of targeted point mutations by incorporating these into the unmarked sequence used in the second transformation step. The use of linear DNA templates (either linearised plasmid or PCR product) in both rounds of transformation ensures that allele replacement is by double-crossover, avoiding problems associated with merodiploid formation/resolution that can arise with suicide vectors. Furthermore, DNA taken up by natural transformation is not prone to degradation by the abundant restriction systems present in *A. pleuropneumoniae*, which can affect efficiency of electroporation [31].

A similar two-step natural transformation method has been described for creating unmarked mutations in *Helicobacter pylori* [32] and *Acinetobacter* sp. strain ADP1 [33], transformable bacteria that do not require specific USS. When creating mutants in *A. pleuropneumoniae* by this method, it is essential to include the 9 bp USS (ACAAGCGGT) required for efficient uptake of DNA by this bacterium [20], [26] in donor DNA used in both steps. To this end, we have generated a *cat-sacB* cassette containing 2 perfect copies of the *A. pleuropneumoniae* USS flanking the *cat* gene. This ensures efficient uptake of the insertion-deletion construct. In the second step, if the unmarked deletion fragment does not contain an endogenous USS, then it can be engineered into primer sequence(s), as we have done.

In this study, we have generated mutations in the highly transformable serovar 15 reference strain HS143 [21], as well as in the serovar 8 clinical isolate MIDG2331. We chose serovar 8 to reflect its high prevalence in the UK [34]. MIDG2331, amenable to this method of creating unmarked deletions, was identified after screening only 15 isolates. In countries where other serovars predominate, we recommend testing a selection of clinical isolates for identification of appropriate transformable strains. Testing must be empirical, as the presence of known competence genes is not sufficient to ensure successful transformation of *A. pleuropneumoniae* strains [20]. Even in *Haemophilus influenzae*, where natural transformation has been extensively studied, the reason for variation in levels of competence of different isolates is not clear [35].

With the availability of whole genome sequences for most serovars of *A. pleuropneumoniae* [36]–[39], it is now possible, using HS143 and/or MIDG2331, to systematically mutate specific highly conserved core genes in order to determine their contribution to the biology and pathogenesis of this bacterium, with a view to improving diagnostics, therapies and vaccine strategies.
